# Oxidative Stress Induces Chondrocyte Apoptosis through Caspase-Dependent and Caspase-Independent Mitochondrial Pathways and the Antioxidant Mechanism of Angelica Sinensis Polysaccharide

**DOI:** 10.1155/2020/3240820

**Published:** 2020-11-07

**Authors:** Chao Zhuang, Su Ni, Zhi-cheng Yang, Rui-ping Liu

**Affiliations:** ^1^Department of Orthopedics, The Affiliated Changzhou No.2 People's Hospital of Nanjing Medical University, Changzhou 213003, China; ^2^Laboratory of Clinical Orthopedics, The Affiliated Changzhou No.2 People's Hospital of Nanjing Medical University, Changzhou 213003, China

## Abstract

**Introduction:**

Chondrocyte apoptosis is considered one of the pathogenic factors of osteoarthritis (OA), but its importance in the pathogenesis of OA remains unclear. Recent research adds progress to the knowledge that the mitochondrial signaling pathway mediates chondrocyte apoptosis in OA.

**Method:**

Rat chondrocyte exposed to H_2_O_2_ was used as the experimental oxidative stress model. Chondrocyte viability was tested by cell counting kit-8 (CCK-8) assay. Cell apoptosis and ROS were tested by flow cytometry. Contents of malondialdehyde (MDA), catalase (CAT), caspase-3, caspase-9, cytochrome C, superoxide dismutase (SOD)-2, and adenosine triphosphate (ATP) were evaluated by biochemical detection. The expressions of related genes and proteins were assessed by quantitative polymerase chain reaction (qPCR) and western blot.

**Results:**

H_2_O_2_ provokes oxidative stress and decreases the viability of chondrocyte, which leads to the release of cytochrome C and inhibition of SOD-2 activity. The damage of mitochondrion disturbs the energy metabolism of chondrocyte and eventually induces chondrocyte apoptosis through the mitochondrial pathway. Furthermore, pretreated with anglicasinensis polysaccharide (ASP) or caspase inhibitors increase the expression of Bcl-2 and Bcl-xL but do not work for the expression of Bax and Bad.

**Conclusion:**

Oxidative stress induces chondrocyte apoptosis through caspase-dependent and caspase-independent mitochondrial pathways. ASP protects chondrocyte from H_2_O_2_-induced oxidative stress and subsequent cell injury through its antioxidant effect by inhibiting the caspase pathway.

## 1. Introduction

OA is a progressive disease of mammalian joints, characterized by destruction of articular cartilage, resulting in discomfort and dysfunction of the affected joints. The stability of articular cartilage depends on the biological activity of chondrocyte, which maintains the stability of cartilage by synthesizing appropriate extracellular matrix (ECM) molecules [1–3]. Chondrocyte is the only cell type in mature cartilage, which is responsible for repairing damaged cartilage tissue. Since articular cartilage is vascularized, the regeneration after injury or degenerative changes is very limited [4]. Although the underlying mechanism has not been fully elucidated, there is considerable evidence that chondrocyte apoptosis is associated with the characteristic cartilage degradation in osteoarthritis (OA) [4–7]. Chondrocyte apoptosis alters the synthesis of the cartilage matrix, leading to cartilage degeneration and destruction, and finally to OA. Accumulated evidence indicates that oxidative stress is involved in chondrocyte apoptosis in OA pathogenesis [8–11].

Reactive oxygen species (ROS) induce oxidative stress, which leads to mitochondrial oxidative damage and cell apoptosis, playing an important role in the pathogenesis of OA [12, 13]. Oxidative stress can interfere with cartilage homeostasis and promote catabolism by inducing apoptosis. Excessive ROS results in mitochondrial dysfunction, which is the main cause of cell injury and death under various pathological conditions [14]. Increased production of ROS was reported to induce cytochrome C releasing from mitochondria and sequentially activate programmed cell death. However, the mechanism of ROS-induced oxidative stress leading to mitochondrial dysfunction and apoptosis in OA chondrocytes remains unclear.

Apoptosis is a complex process characterized by nuclear chromatin condensation, DNA breakage, cell contraction, and apoptotic body formation [15, 16], including intrinsic pathways dependent on mitochondria and extrinsic pathways regulated by death receptors on the cell surface in mammalian cells [17]. A wide range of signals can trigger the intrinsic pathway, including oxidative stress [6, 18]. Chondrocyte apoptosis ultimately leads to the failure of cartilage matrix renewal, as chondrocyte is the only resident cell responsible for synthesizing and maintaining ECM molecules in articular cartilage [3, 4, 10]. Therefore, it is necessary to elucidate the molecular pathway of chondrocyte apoptosis in OA [19].

Although oxygen can diffuse into articular cartilage, chondrocyte has mitochondria and respiratory function *in vitro*, the pathogenesis of mitochondrial-mediated OA has not been extensively studied, because articular chondrocyte must survive in an avascular and hypoxic environment to maintain the tissue integrity, which requires an adaptive increase of anaerobic glycolysis to support adenosine triphosphate (ATP) synthesis [20–22]. However, chondrocyte mitochondrial damage affects ATP production, which directly reduces the synthesis of cartilage matrix and enhances matrix calcification [15, 23, 24]. The mitochondrial function of normal chondrocyte plays an important role in ATP reserve in response to oxidative stress during the development of OA. Studies have shown that mitochondrial dysfunction is related to ROS. Mitochondria may be the first target of oxidative stress. Mitochondria themselves are sources of ROS [25–27]. If this is the case, mitochondria may also be the central control point of apoptosis induced by oxidative stress.

We previously observed that anglicasinensis polysaccharide (ASP), as an antioxidant, inhibits hydrogen peroxide (H_2_O_2_)-mediated oxidative stress injury in both rat chondrocyte and human chondrocyte, but we did not explore its specific mechanism. Canonical cell apoptosis is considered to depend on caspase activation, and we have previously demonstrated that H_2_O_2_ acts as a mediator of cell apoptosis *in vitro* [28]. Here, we further demonstrate the change of B-cell lymphoma (Bcl)-2family in this model of cell apoptosis and provide evidence that oxidative stress drives cell apoptosis through caspase-dependent and caspase-independent mitochondrial pathways, which retain key features of cell apoptosis. We also found that ASP resists apoptosis induced by oxidative stress by inhibiting the caspase pathway.

## 2. Materials and Methods

### 2.1. Reagents

Collagenase II was obtained from Sigma-Aldrich (St Louis, MO, USA). Dulbecco's modified Eagle's medium (DMEM) with 100 U penicillin and 100 *μ*g streptomycin and fetal bovine serum (FBS) were from Gibco™ (Grand Island, NY, USA). FITC Annexin V Apoptosis Detection Kit was from BD PharmingenTM (San Diego, CA, USA). Cell Counting Kit-8 (CCK-8), 0.25% trypsin, ROS assay kit, malondialdehyde (MDA) assay kit, catalase (CAT) assay kit, caspase-9 and caspase-3 colorimetric assay kits, cytochrome C ELISA kit, superoxide dismutase (SOD)-2 assay kit, ATP assay kit, and BCA Protein assay kit were purchased from Beyotime Biotechnology (Shanghai, China). TRIzol was from Invitrogen (Carlsbad, CA, USA). High Capacity cDNA Reverse Transcription kit was obtained from Applied Biosystems (Foster City, CA, USA). SYBR® Select Master Mix was obtained from Applied Biosystems (Austin, TX, USA). Collagenase II was dissolved in DMEM and diluted to 2 mg/mL to digest articular cartilage. Caspase inhibitor (Ac-DEVD-FMK) was purchased from Cell Signaling Technology (Danvers, MA, USA) and dissolved in dimethyl sulfoxide (DMSO). ASP was purchased from Shanghai Yilin Biotech. Co., Ltd (Shanghai, China). The purity of ASP is above 90%. The component sugars are glucose, galactose, arabinose, rhamnose, mannose, and xylose. The average molecular weight of ASP was 85.0 kDa.

### 2.2. Cell Harvest and Culture

A modified method for harvesting chondrocyte was conducted as described previously [29]. In brief, chondrocytes were isolated from the articular cartilage of three-week-old male Sprague-Dawley rats. The cartilage was removed from animals that were subsequently euthanized via an overdose of anesthesia. The cartilage was cut into thin slices, washed with sterile phosphate-buffered saline (PBS), and then digested with 2 mg/mL collagenase type II in DMEM overnight at 37°C within the incubator. The digested cartilage was collected and centrifuged. The pellets were resuspended in DMEM and filtered through a 70 *μ*m nylon cell strainer (FALCON, Pittsburgh, PA, USA). The primary chondrocytes were cultured in DMEM supplemented with 10% FBS in a 5% CO_2_ incubator at 37°C. Confluent chondrocytes were split in 1 : 3 ratios up to passages 2-3 and used for subsequent experiments. This study was reviewed and approved by the ethics committee of the Second People's Hospital of Changzhou, Jiangsu, China.

### 2.3. Cell Viability Assay

To assess the time-response relationship of H_2_O_2_ in the experiment, the cell viability was evaluated by CCK-8 assay. Chondrocytes were plated in 96-well plates at a density of 5 × 10^3^ cells/well to adhere overnight and treated with 0.3 mM H_2_O_2_ for 0 h, 0.5 h, 1 h, 2 h, 4 h, 12 h, and 24 h. After incubation time, 10 *μ*L of CCK-8 solution was added to each well and further incubated for 1 h at 37°C in 5% CO_2_. Absorbance at 450 nm-650 nm was measured using a microplate reader (SpectraMax Plus 384, MD, USA).

### 2.4. Cell Treatment

Cultured chondrocytes were divided into four groups and treated with various treatments. Group 1: chondrocytes cultured in DMEM supplemented with 10% FBS were used as normal control; Group 2: cultured chondrocytes were treated with 0.3 mM H_2_O_2_ for 0.5 h; Group 3: cultured chondrocytes were pretreated with 10 mM ASP for 1 h and then incubated with 0.3 mM H_2_O_2_ for 0.5 h; Group 4: cultured chondrocytes were pretreated with 10 *μ*M Ac-DEVD-FMK for 1 h and then incubated with 0.3 mM H_2_O_2_ for 0.5 h.

### 2.5. Cell Apoptosis Detection

To quantify the percentage of chondrocyte undergoing apoptosis, the FITC Annexin V Apoptosis Detection Kit was used according to the manufacturer's instructions. Briefly, after treatment, chondrocytes were harvested and washed twice with cold PBS, then resuspended in 100 *μ*L binding buffer into which 5 *μ*L of FITC Annexin V and 5 *μ*L propidium iodide (PI) were added for 15 min at 25°C in the dark. After incubation, 400 *μ*L binding buffer was added, and chondrocytes were analyzed with a FACScan flow cytometer (BD Biosciences, San Jose, CA, USA).

### 2.6. The Measurement of ROS Production in Chondrocyte

The ROS level in chondrocyte was measured by using a commercialized kit according to the manufacturer's optimized instructions. Briefly, the number of washed chondrocytes was counted using a cell-count board. They were suspended in diluted DCFH-DA (10 mM) in PBS and incubated at 37°C for 20 min. After washing twice with PBS, ROS production in the resuspended solution was measured by using a FACScan flow cytometer (BD Biosciences, San Jose, CA, USA).

### 2.7. Measurement of MDA Production and CAT Activity

Treated chondrocytes were washed twice with cold PBS, and total proteins were extracted with 100 *μ*l RIPA for 15 min. Lysates were processed under ultrasonic and centrifuged at 13000 g for 5 min. The supernatants were quantified with BCA Protein Assay. MDA equivalents and CAT levels were measured with a commercially available kit according to the manufacturer's instructions.

### 2.8. Determination of Total Cytochrome C

Total cytochrome C was determined by cytochrome C ELISA kit according to the manufacturer's instructions. In brief, chondrocytes were washed three times in PBS and then were resuspended in Cell Lysis Buffer to a concentration of 1.5 × 10^6^ cells/mL. Chondrocytes were incubated for 1 h at room temperature with gentle mixing and then centrifuged at 1000 g for 15 min. The supernatant was used for determination of cytochrome C. 50 *μ*L of calibrator diluent was added to each well of a 96-well plate. Then, 50 *μ*L of standards, control, and samples were added, respectively. Covered with an adhesive strip, the plate was then incubated for 0.5 h at 37°C. After incubation, the plate was washed five times with washing buffer, and 50 *μ*L of cytochrome C conjugate was added to each well with 0.5 h incubation at 37°C. Repeat the washing steps. 100 *μ*L of substrate solution was added to each well, and the plate was incubated for 10 min at 37°C avoiding of light. 50 *μ*L of stop solution was added to each well. Optical density was finally determined within 30 min, and the absorbance at 450 nm was measured using a microplate reader (SpectraMax Plus 384, MD, USA).

### 2.9. SOD-2 Activity Detection

The enzyme activity of SOD-2 was determined using an assay kit with WST-8 (2-(2-methoxy-4-nitrophenyl)-3-(4-nitrophenyl)-5-(2, 4-disulfophenyl)-2H-tetrazolium, monosodium salt) according to the manufacturer's instructions. Briefly, SOD-1 inhibitors A and B were successively added to the samples to inhibit residual SOD-1 activity for 60 min at 37°C and then mixed with WST-8 enzyme working solution for 30 min at 37°C. The absorbance at 450 nm was measured using a microplate reader (SpectraMax Plus 384, MD, USA).

### 2.10. Assay of ATP

The ATP content was determined by a luciferin/luciferase method using an ATP assay kit according to the manufacturer's instructions. In brief, chondrocytes were centrifuged at 12,000 g for 5 min at 4°C to prepare the supernatants for ATP testing. An ATP concentration standard curve was generated using a series of known concentrations of ATP standard solutions. Subsequently, 100 *μ*L ATP assay buffer was added to each well, incubated for 3 min, and then mixed with 20 *μ*L of supernatant. Luminescence measurement was using a luminometer. After calibration with the standard curve, the ATP content of the samples was determined.

### 2.11. Measurement of Caspase-9 and Caspase-3 Activities

Caspase-9 and caspase-3 activities were measured by colorimetric assay kits according to the manufacturer's instructions. Briefly, cells were collected and lysed using the lysis buffer provided. The caspase-9 and caspase-3 activity colorimetric assays are based on the hydrolysis of the peptide substrate acetyl, resulting in the release of p-nitroaniline moiety, which has a high absorbance at 405 nm that was detected by a microplate reader (SpectraMax Plus 384, MD, USA).

### 2.12. Quantitative Real-Time Reverse Transcription Polymerase Chain Reaction (qRT-PCR)

Total RNA was extracted from treated samples using TRIzol. High Capacity cDNA Reverse Transcription kit was used to reverse transcribe total RNA (1 *μ*g) according to the manufacturer's protocol. Bcl-2, Bcl-XL, Bax, Bad, and glyceraldehyde 3-phosphate dehydrogenase (GAPDH) were amplified using SYBR® Select Master Mix in a Bio-Rad iQ5. The specific primer sequences (designed by Sangon Biotech. Co., Ltd, Shanghai, China) are presented in Table 1. The data were calculated by the comparative threshold cycle method.

### 2.13. Western Blot

Chondrocytes were harvested and lysed in RIPA buffer for total protein extraction. The protein concentration of each sample was determined by the BCA Protein Assay kit. Equal amounts of protein (10 *μ*g) were boiled and subjected to electrophoresis on 10% sodium dodecyl sulfate-polyacrylamide gels and transferred to a polyvinylidene fluoride (PVDF) membrane. After being blocked for 1 h in tris buffered saline with Tween-20 with 5% nonfat milk, the PVDF membrane was then probed with Rabbit anti-rat Bcl-2 antibody, rabbit anti-rat Bcl-XL antibody, rabbit anti-rat Bax, rabbit anti-rat Bad antibody, and rabbit anti-rat GAPDH antibody (all these antibodies were purchased from Cell Signaling Technology, Danvers, MA, USA and diluted in 1 : 1000) overnight at 4°C and then with horseradish peroxidase-conjugated secondary antibodies for 1 h at room temperature. The blots were detected with the enhanced chemiluminescence assay kit. Rabbit anti-rat GAPDH antibody was used to detect GAPDH signal as an internal loading control, and relative expression levels were quantified by running the Quantity One software (Bio-Rad Laboratories, Hercules, CA, USA).

### 2.14. Statistical Analysis

Data shown in our study were represented as means ± SD from at least three independent experiments. One-way ANOVA followed by a student's *t*-test was conducted for comparison between the two groups. Significant difference was considered when *P* < 0.05.

## 3. Results

### 3.1. H_2_O_2_ Impaired the Viability of Chondrocyte by Time

**Figure 1:**
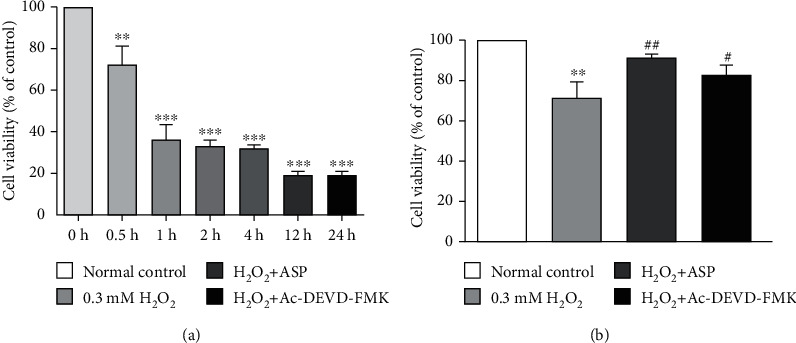
Cell viability in chondrocytes. (a) Chondrocytes were treated with 0.3mMH_2_O_2_ for 0 h, 0.5 h, 1 h, 2 h, 4 h, 12 h, and 24 h. Cell viability was detected by CCK-8. (b) Chondrocytes were induced by various treatments. Cell viability was detected by CCK-8. Results are presented as means ± standard deviation of three independent experiments. Untreated chondrocytes were used as normal control and considered 100% viable. ^∗∗^*P* < 0.01, ^∗∗∗^*P* < 0.001 versus normal control. ^#^*P* < 0.05, ^##^*P* < 0.01 versus H_2_O_2_ group.

In our previous research, we examined the concentration effect of H_2_O_2_ on chondrocyte viability in rats [28]. In the present study, we tested the effects of different time duration of H_2_O_2_ on chondrocyte. Cell viability was measured at different time points after H_2_O_2_ treatment. The results showed that the viability of chondrocyte decreased by 30%, 63%, 67%, and 68% after 0.5 h, 1 h, 2 h, and 4 h of H_2_O_2_ treatment, respectively. With the prolongation of the time, the damage gradually aggravated, and the viability of chondrocyte decreased by 80% in 12 hours or 24 hours (Figure 1(a)). As we expected, H_2_O_2_ induces oxidative stress and reduces cell viability. When pretreated with ASP or caspase inhibitor, cell viability decreased slightly compared with the control group (*P* > 0.05) and increased by 25% and 18% compared with H_2_O_2_ group (Figure 1(b)), suggesting that ASP may protect chondrocyte from the oxidative stress damage induced by H_2_O_2_ through caspase passway.

### 3.2. H_2_O_2_ Significantly Increased Chondrocyte Apoptosis

**Figure 2:**
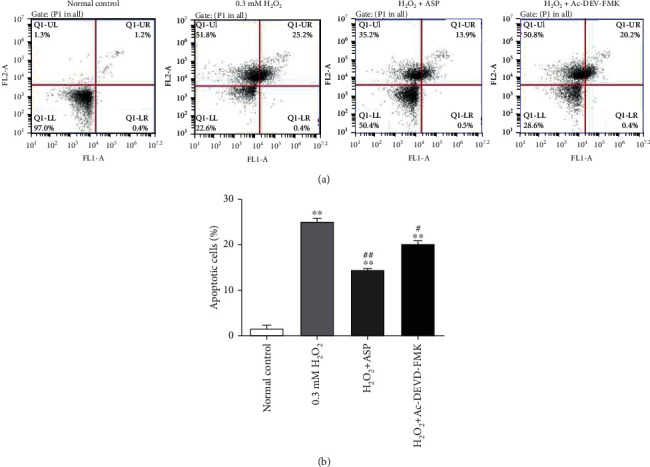
Cell apoptosis in chondrocytes. (a) Chondrocytes were induced by various treatments. Chondrocytes cultured in DMEM for 0.5 h was used as a normal control. FITC Annexin V/PI staining and flow cytometry assays were used to detect cell apoptosis. (b) Results of cell apoptosis in different groups. Results are presented as means ± standard deviation of three independent experiments. ^∗∗^*P* < 0.01 versus normal control. ^#^*P* < 0.05, ^##^*P* < 0.01 versus H_2_O_2_ group.

The apoptosis of chondrocyte was detected by flow cytometry assay (Figure 2(a)). Chondrocyte cultured in DMEM was used as a negative control. The percentage of apoptotic cells in quadrants Q1-UR and Q1-LR was calculated and shown in Figure 2(b). When treated with H_2_O_2_, the apoptotic rate of chondrocyte increased by about 25 times, suggesting that H_2_O_2_ can induce oxidative stress and promote cell apoptosis. When pretreated with ASP or caspase inhibitor, the apoptotic rate increased by 14 times and 20 times, respectively, compared with the control group, and decreased by 44% and 20%, respectively, compared with H_2_O_2_ group. These results indicate that ASP may inhibit the chondrocyte apoptosis induced by H_2_O_2_ and probably through the caspase passway.

### 3.3. The Extent of Oxidative Stress as Measured by the Level of Intracellular ROS, MDA, and CAT

**Figure 3:**
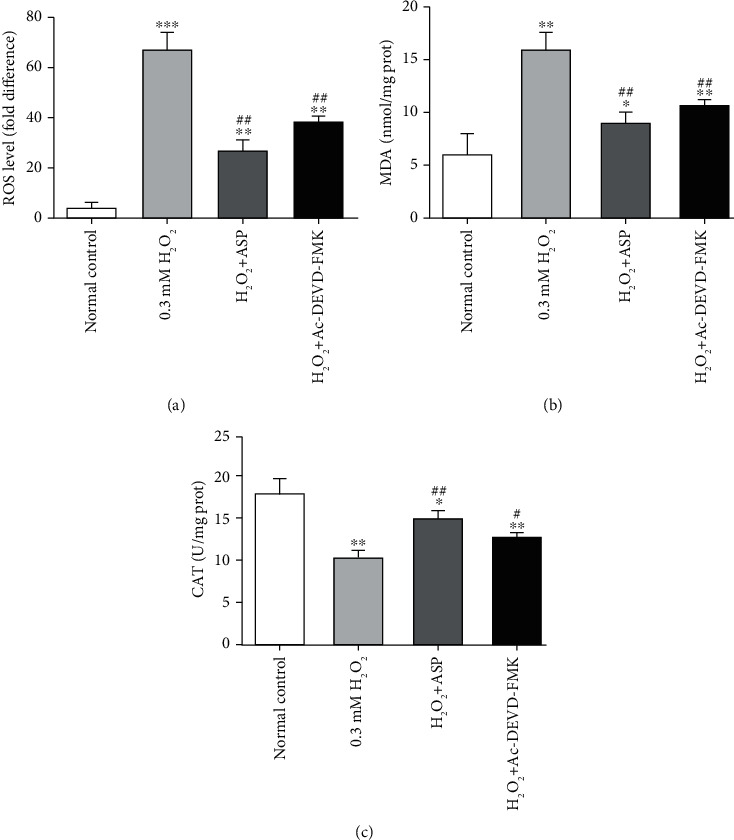
The detection of oxidative stress in different groups. (a) Cultured and treated chondrocytes were harvested, and the levels of intracellular ROS in different groups were analyzed with a FACScan flow cytometer. (b) Cultured and treated chondrocytes were harvested, total proteins were extracted, and the levels of MDA equivalents were detected by assay kit. (c) Cultured and treated chondrocytes were harvested, total proteins were extracted, and the levels of CAT were detected by assay kit. Results are presented as means ± standard deviation of three independent experiments. ^∗^*P* < 0.05, ^∗∗^*P* < 0.01, ^∗∗∗^*P* < 0.001 versus normal control. ^#^*P* < 0.05, ^##^*P* < 0.01 versus H_2_O_2_ group.

The level of intracellular ROS, MDA, and CAT in chondrocyte was considered as indicators of oxidative stress. After treatment with H_2_O_2_, intracellular oxidative stress was increased; the levels of intracellular ROS and MDA were 13.34 times and 2.59 times higher than that of the control group, respectively (Figures 3(a) and 3(b)). When pretreated with ASP or caspase inhibitor, the measurement of intracellular ROS increased by 5.16 times and 7.56 times, respectively, as compared with the control group but decreased by 60% and 44%, respectively, compared with H_2_O_2_ group (Figure 3(a)). Meanwhile, the measurement of MDA increased by 1.44 times and 1.74 times, respectively, as compared with the control group but decreased by 45% and 33%, respectively, compared with H_2_O_2_ group (Figure 3(b)). After H_2_O_2_ treatment, the activity of CAT decreased by 43% compared with the control group. When pretreated with ASP or caspase inhibitor, the CAT activity decreased by 17% and 30%, respectively, compared with the control group but increased 1.44 times and 1.23 times than that in H_2_O_2_ group, respectively (Figure 3(c)). The results suggest that ASP may suppress the production of intracellular ROS and MDA and upregulate the activity of CAT by inhibiting oxidative stress through caspase pathway.

### 3.4. Mitochondrial Function Was Hampered by H_2_O_2_ via Changing the Contents of Cytochrome C, SOD-2, and ATP

**Figure 4:**
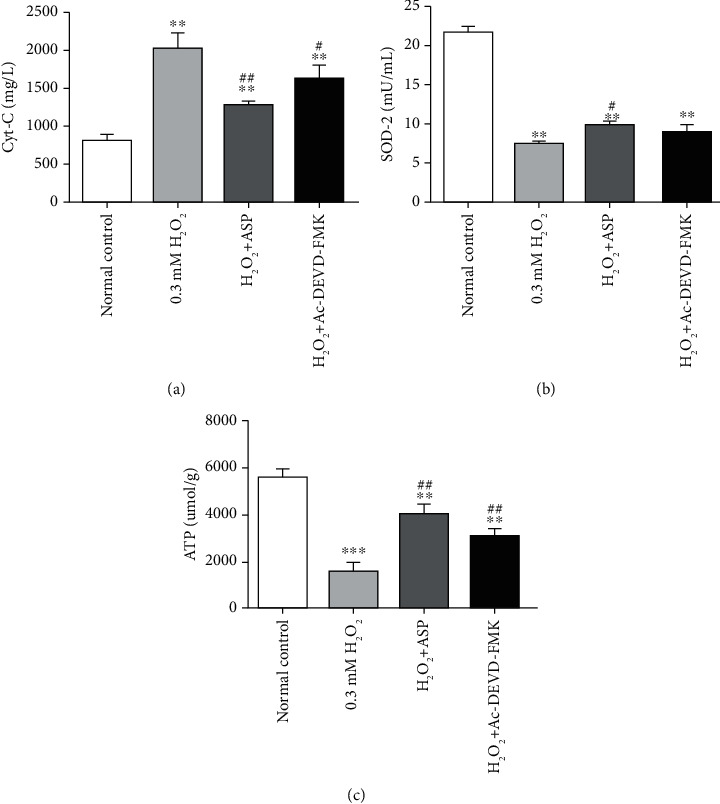
Alter of mitochondrial function in chondrocytes induced by various treatments. Cultured and treated chondrocytes were harvested, and the levels of cytochrome C (a), SOD-2 (b), and ATP (c) in different groups were measured by assay kit. Results are presented as means ± standard deviation of three independent experiments. Untreated chondrocytes were used as normal control. ^∗∗^*P* < 0.01, ^∗∗∗^*P* < 0.001 versus normal control. ^#^*P* < 0.05, ^##^*P* < 0.01 versus H_2_O_2_ group.

Cytochrome C, SOD-2, and ATP were considered as indicators reflecting mitochondrial function. After adding H_2_O_2_, mitochondrial function was damaged by oxidative stress, which led to the overflow of cytochrome C. So, cytochrome C was 2.5 times higher than that of the control group. When pretreated with ASP or caspase inhibitor, the cytochrome C value increased by 1.6 times and 2 times, respectively, as compared with the control group but decreased by 20% and 36%, respectively, compared with H_2_O_2_ group (Figure 4(a)). After treatment with H_2_O_2_, the increase of oxidative stress led to the decrease of antioxidant enzyme activity. The activity of SOD-2 decreased by 65% compared with the control group. When pretreated with ASP or caspase inhibitor, the SOD-2 activity decreased by 50% and 58%, respectively, compared with the control group. SOD-2 activity in H_2_O_2_+ASP group was 25% higher than that in H_2_O_2_ group (*P* < 0.05), while SOD-2 activity in H_2_O_2_+Ac-DEVD-FMK group was not significantly higher than that in H_2_O_2_ group (*P* > 0.05) (Figure 4(b)). After H_2_O_2_ treatment, excessive oxidative stress increased energy consumption, impaired mitochondrial function, and reduced energy production. ATP level of chondrocyte decreased by 71% compared with the control group. When pretreated with ASP or caspase inhibitor, ATP level decreased by 28% and 44%, respectively, compared with the control group but increased 2.5 times and 2.0 times, respectively, compared with H_2_O_2_ group (Figure 4(c)). The results show that ASP could protect the integrity of mitochondrial function, reduce the overflow of cytochrome C, increase the activity of SOD-2, and improve energy metabolism. The mechanism may be through the caspase pathway.

### 3.5. Caspase Activity in Oxidative Stress-Induced Chondrocyte Apoptosis

**Figure 5:**
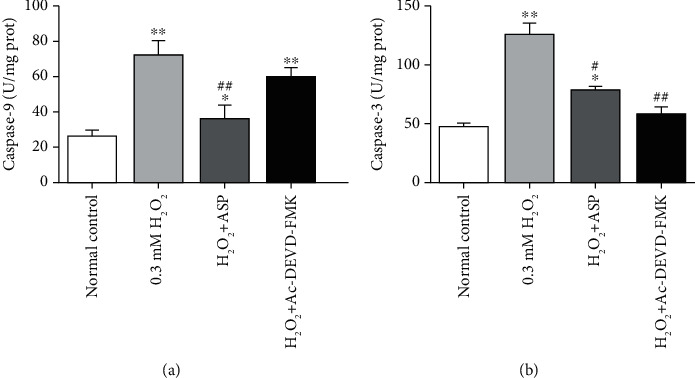
The measurement of caspases activities in different groups. Cultured and treated chondrocytes were harvested, total proteins were extracted, and the levels of cleaved caspases were detected by assay kit. Results are presented as means ± standard deviation of three independent experiments. ^∗^*P* < 0.05, ^∗∗^*P* < 0.01 versus normal control. ^#^*P* < 0.05, ^##^*P* < 0.01 versus H_2_O_2_ group.

Caspase is active only when it is cracked, so cleaved caspases were measured by colorimetric assay kits. After treatment with H_2_O_2_, the levels of caspase-9 and caspase-3 were increased by 2.78 folds and 2.69 folds than that of the control group, respectively (Figures 5(a) and 5(b)). When pretreated with ASP or caspase inhibitor, the measurement of caspase-9 increased by 1.36 folds and 2.29 folds, respectively, as compared with the control group but decreased by 50% and 17%, respectively, compared with H_2_O_2_ group (Figure 5(a)). Meanwhile, the measurement of caspase-3 increased by 1.69 folds and 1.23 folds, respectively, as compared with the control group but decreased by 40% and 55%, respectively, compared with H_2_O_2_ group (Figure 5(b)). The results suggest that ASP can inhibit oxidative stress through caspase pathway.

### 3.6. Expressions of Bcl-2 Genes

**Figure 6:**
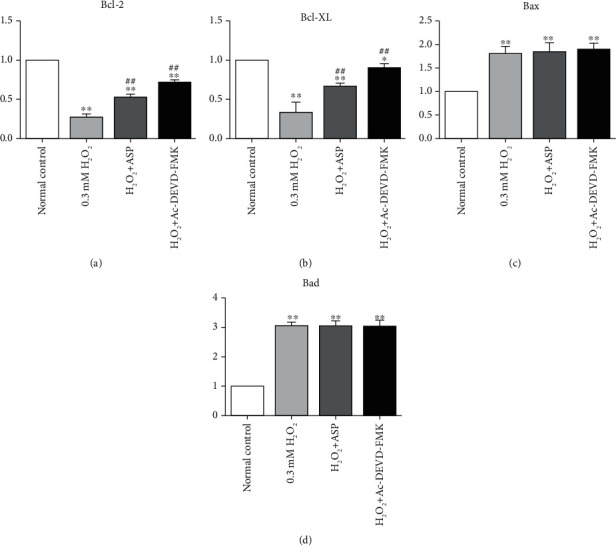
Expression of related genes in mitochondrial pathways. Cultured and treated chondrocytes were harvested and total RNA was extracted, followed by qRT-PCR for detection of relative gene expression levels. Results are presented as mean ± standard deviation of three independent experiments. Untreated chondrocytes were used as normal control. ^∗^*P* < 0.05, ^∗∗^*P* < 0.01 versus normal control. ^##^*P* < 0.01 versus H_2_O_2_ group.

The mRNA expression of Bcl-2 and Bcl-XL was downregulated (decreased by 73% and 66%, respectively) in chondrocyte treated with H_2_O_2_ (Figures 6(a) and 6(b)), while the mRNA expression of Bax and Bad was upregulated (increased 1.8 times and 3.07 times, respectively)(Figures 6(c) and 6(d)), compared with normal control. When pretreated with ASP or caspase inhibitor, the mRNA expression of Bcl-2 and Bcl-XL was still lower than that of the control group (decreased by 48% and 29% in Bcl-2 and decreased by 34% and 11% in Bcl-XL, respectively). However, ASP or caspase inhibitor alleviated the inhibition of Bcl-2 and Bcl-XL compared with H_2_O_2_ group (increased 1.92 times and 2.62 times in Bcl-2 and increased 1.96 times and 2.67 times in Bcl-XL, respectively) (Figures 6(a) and 6(b)). The mRNA expression of Bax and Bad was higher than that of the control group after adding ASP or caspase inhibitor (increased 1.85 times and 1.91 times in Bax and increased 3.08 times and 3.07 times in Bad, respectively), but there was no significant difference compared with H_2_O_2_ group (*P* > 0.05)(Figures 6(c) and 6(d)). The results showed that the mechanism of ASP protecting mitochondrial functional integrity was achieved by increasing the expression of apoptosis inhibiting genes, but it could not change the expression of apoptosis promoting genes, which was consistent with the effect of caspase inhibitor.

### 3.7. Expressions of Bcl-2 Proteins

**Figure 7:**
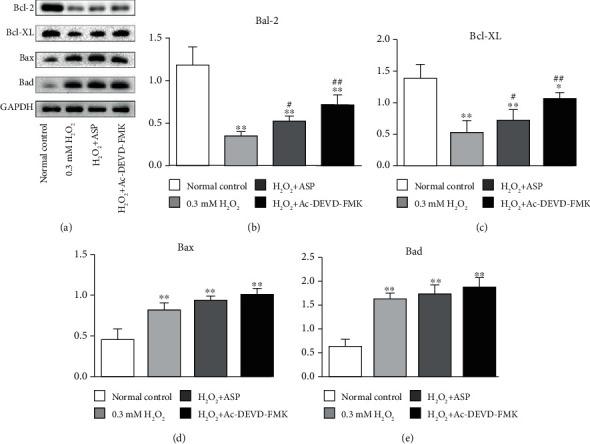
Oxidative stress activates caspase-dependent and caspase-independent mitochondrial pathways in chondrocyte apoptosis. Following treatment, the levels of Bcl-2, Bcl-XL, Bax, and Bad in total cell lysates were determined by western blot analysis. Representative western blot (a) and quantification data (b–e) are shown, respectively. The relative protein levels were normalized to the level of the internal control, GAPDH, and presented as fold changes relative to the control group (the level of the control group was set as 1). Results are presented as mean ± standard deviation of three independent experiments. Untreated chondrocytes were used as normal control. ^∗^*P* < 0.05, ^∗∗^*P* < 0.01 versus normal control. ^#^*P* < 0.05, ^##^*P* < 0.01 versus H_2_O_2_ group.

To determine the mitochondrial pathway by which H_2_O_2_ induced chondrocyte apoptosis, the expressions of mitochondrial-associated proteins were studied by western blot analysis (Figure 7(a)). Compared with the control group, the protein levels of Bcl-2 and Bcl-XL decreased by 69% and 62%, respectively, after adding H_2_O_2_. When pretreated with ASP or caspase inhibitor, the protein levels were still lower than that of the control group (decreased by 59% and 41% in Bcl-2 and decreased by 48% and 22% in Bcl-XL, respectively), but higher than that of H_2_O_2_ group (increased 1.32 times and 1.91 times in Bcl-2 and increased 1.35 times and 2 times in Bcl-XL, respectively) (Figures 7(b) and 7(c)). The protein levels of Bax and Bad increased 1.8 times and 2.79 times, respectively, after adding H_2_O_2_. After adding ASP or caspase inhibitor, the protein levels increased significantly compared with the control group (increased 2 times and 2.02 times in Bax and increased 2.8 times and 2.93 times in Bad, respectively), but there was no significant difference compared with H_2_O_2_group (*P* > 0.05) (Figures 7(d) and 7(e)). The results showed that ASP could increase the level of mitochondrial antiapoptosis proteins but could not change the level of mitochondrial proapoptosis proteins, which further suggested that the antioxidant function of ASP depended on caspase signaling pathway.

## 4. Discussion

H_2_O_2_ is formed by superoxide anion through superoxide dismutase and has many biological effects on various cells. H_2_O_2_ produces ROS, which is closely related to chondrocyte apoptosis *in vivo* and *in vitro*. Compared with other ROS sources, H_2_O_2_ has lower biological activity, but much higher ability of transmembrane diffusion, which makes it an ideal signal transduction molecule. It is also believed to regulate a variety of cellular functions and induce apoptosis of various cells, including chondrocyte, at a low dose [30]. Therefore, H_2_O_2_ was used in this oxidative stress model in chondrocyte to reveal the mechanisms of oxidative stress-induced cell apoptosis in OA.

In previous research, we confirmed that caspase-3 and caspase-9 participated in the H_2_O_2_-induced chondrocyte apoptosis by western blot [28]. In this experiment, we used a caspase inhibitor and found that the viability of chondrocyte increased significantly compared to the H_2_O_2_ group (Figure 1(b)). The result was consistent with the detection of apoptosis (Figure 2) and further confirmed that caspases are indeed involved in the apoptosis of chondrocyte induced by oxidative stress. We further used ASP as a therapeutic drug. After pretreatment with ASP, we found the same effect of caspase inhibitor on chondrocyte activity and apoptosis (Figures 1(b) and 2), which suggested that the antioxidant capacity of ASP was related to the inhibition of caspase pathway.

Oxidative stress is a cell injury caused by oxygen free radicals, which is the main cause of cartilage injury [31]. Oxidative stress plays an important role in the degradation and oxidation of cartilage ECM and triggers apoptosis of chondrocyte, which is considered to be the common molecular basis for the initiation and subsequent progress of OA. During the development of OA, chondrocytes produce excessive ROS, owing to the destruction of which, the degeneration of chondrocyte coexists with oxidative stress [4, 7, 32]. In our experiments, we chose intracellular ROS, MDA, and CAT as indicators of oxidative stress. The results showed a biochemical imbalance caused by excessive production of ROS with the increase of MDA level and decrease of CAT activity (Figure 3), which is consistent with other research [17, 25]. When pretreated with ASP, the results were consistent with our previous research [12, 29], which further confirmed the antioxidant capacity of ASP. In the present research, we chose caspase inhibitor as a positive reference, and the results showed that the trend of remission effect of ASP was the same as that of caspase inhibitor. This indicates the mechanism of ASP, which is not involved in previous studies.

As reported, mitochondrial functional integrity is compromised by oxidative stress when H_2_O_2_ changes the permeability of the mitochondrial membrane and releases cytochrome C into the cytoplasm [22, 33]. Therefore, in the process of H_2_O_2_-induced oxidative stress, the cytochrome C level is elevated (Figure 4(a)). When using caspase inhibitor, due to the interruption of apoptotic process and feedback regulation, the detected value of cytochrome C is lower (Figure 4(a)). Pretreatment with ASP before incubation with H_2_O_2_ also reduced the release of cytochrome C, which further suggested that the protective effect of ASP was related to the caspase pathway (Figure 4(a)). Most of the ROS in cells are produced in mitochondria. To counteract this process, cells have many defense mechanisms that help detoxify ROS [34, 35]. Endogenous antioxidants such as SOD are important biomarkers of the antioxidant systems. These endogenous antioxidants can inhibit the excessive production of ROS and avoid excessive lipid peroxidations. SOD is a key element in removing ROS from the human body, which can prevent ROS from damaging cells. SOD is a detoxifying enzyme that converts superoxide to H_2_O_2_ and then to water [30, 33]. SOD-2 is the main and usually only SOD in the mitochondrial matrix. Because mitochondria are the main source of superoxide in cells and superoxide is not able to pass through the cell membrane, SOD-2 may be the most critical SOD to reduce superoxide-induced injury in cells [13, 14]. In the experiment, we found that the activity of SOD-2 was significantly inhibited by H_2_O_2_. With the progress of oxidative stress, the activity of SOD-2 decreased significantly (Figure 4(b)). The experimental results are consistent with the previous literature, which further clarifies the importance of SOD-2. We speculate that if one of the apoptotic links can be interrupted, SOD-2 activity should be able to recover. When caspase inhibitor or ASP were used, SOD-2 activity increased to varying degrees as expected, which further confirmed that caspase signaling pathway was indeed involved in the regulation of mitochondrial apoptotic pathway and ASP played a similar role as caspase inhibitor (Figure 4(b)). Mitochondria are not only the target of ROS but also the main source of ROS. It can be imagined that the production of ROS mediated by mitochondria under oxidative stress leads to chondrocyte apoptosis. ROS can directly change mitochondrial permeability, resulting in loss of mitochondrial membrane potential, reduction of ATP production [18, 36, 37], which was further confirmed in our experiments (Figure 4(c)). When pretreated with caspase inhibitor or ASP, apoptosis was ameliorated to a certain extent, ATP production increased (Figure 4(c)). It is suggested that the ability of ASP to protect the integrity of mitochondrial and improve energy metabolism is achieved by inhibiting endogenous apoptosis through the caspase pathway.

Bcl-2 family is mainly located on the outer membrane of mitochondria, which are the main regulator and effector of stress-induced intrinsic apoptotic pathway [26, 33]. The Bcl-2-related protein is characterized by the presence of one or more of four conserved Bcl-2 homology (BH1–BH4) domains. Based on their structure and function, Bcl-2 family members divide into antiapoptotic members (such as Bcl-2 and Bcl-XL), multidomain proapoptotic members (such as Bax), and BH3-only pro-apoptotic members (such as Bad). Induction of apoptosis is regulated by the expression of Bcl-2 and Bcl-XL through maintenance of cytochrome C release from the mitochondria. Released cytochrome C activates apoptotic protease-activating factor-1, which oligomerizes to form an apoptosome. This structure, in turn, recruits and activates caspase-9. Activated caspase-9 cleaves and activates executioner caspases, such as caspase-3, and eventually results in apoptosis [13, 28]. Bcl-2 and Bcl-XL reside on the cytoplasmic face of the outer mitochondrial membrane, which is involved in the regulation of chondrocyte apoptosis in osteoarthritic cartilage. Both of these proteins play a crucial role in the maintenance of mitochondrial membrane integrity by preventing the activation of proapoptotic proteins Bax and Bad. Bax is well known as a proapoptotic protein that can translocate to the mitochondria from the cytoplasm and that affects the release of cytochrome C from mitochondria. Bad is a BH-3 protein bridging the mitochondrial pathway with apoptosis signals. The increased Bad thereafter bonded to and activated the Bax. Once the Bax dimerized and activated, they permeabilized the mitochondria outer membrane, the released intermembrane space contained proteins. Therefore, these proteins regulate the apoptotic process principally via the mitochondrial pathway [7, 20, 32]. When pretreated with caspase inhibitor, we detected the corresponding changes of caspase-3 activity (Figure 5(b)) and the levels of Bcl-2 and Bcl-XL genes and proteins (Figures 6 and 7), but no significant changes in the levels of Bax and Bad genes and proteins (Figures 6 and 7). This finding suggested that mitochondrial antiapoptotic proteins were regulated by the caspase signaling pathway directly, while proapoptotic proteins were not regulated by the caspase signaling pathway directly. Ac-DEVD-FMK inhibited chondrocyte apoptosis to some extent by blocking executioner caspase-3 activity, protecting mitochondrial function, increasing Bcl-2 and Bcl-XL contents, but had no influence on Bax and Bad. After oxidative stress, Bax and Bad proteins activity increased, mitochondrial function was destroyed, and chondrocyte apoptosis was promoted. The contents of Bax and Bad could not be decreased by ASP pretreatment either (Figures 6 and 7), which was similar to that of caspase inhibitor, which further proved that the antioxidation of ASP was closely related to the caspase pathway.

## 5. Conclusion

This research is based on previous experiments to study the changes of mitochondrial function and the activation of apoptotic pathways involved in the process of oxidative stress-induced chondrocyte apoptosis. We found that caspase-dependent and caspase-independent mitochondrial pathways both take part in the regulation of H_2_O_2_-induced chondrocyte apoptosis. We further confirmed that the antioxidant capacity of ASP is achieved by inhibiting the caspase pathway.

## Figures and Tables

**Table 1:** Primer sequences for qRT-PCR.

Gene	Forward (5′→3′)	Reverse (5′→3′)
Bcl-2	GCTCAGCCCTGTGCCACCTG	CAGAGGTCGCATGCTGGGGC
Bcl-XL	CGGCTCTCGGCTGCTGCATT	CGGGGCACTGTGCGTGGAAA
Bax	ACTTCAACTGGGGCCGCGTG	GAGGCCTTCCCAGCCACCCT
Bad	CGACAGTCTCAGGAGGAACC	CCTTCTCCATACCAGACGGA
GAPDH	TGAACGGGAAGCTCACTGG	TCCACCACCCTGTTGCTGTA

## Data Availability

Data available on request.
